# Changes in Elder Mistreatment Risk Factors Reported by Caregivers of Older Adults During the COVID-19 Pandemic

**DOI:** 10.1093/geroni/igab046.280

**Published:** 2021-12-17

**Authors:** Lena Makaroun, Scott Beach, Tony Rosen, Ann-Marie Rosland

**Affiliations:** 1 University of Pittsburgh, Pittsburgh, Pennsylvania, United States; 2 University of Pittsburgh, University of Pittsburgh, Pennsylvania, United States; 3 Weill Cornell Medical College / NewYork-Presbyterian Hospital, PELHAM, New York, United States

## Abstract

This study aimed to assess how the COVID-19 pandemic has impacted caregiver (CG)-related risk factors for elder mistreatment (EM) in a community sample of CGs. A non-probability sample of 433 CGs caring for care recipients (CRs) age ≥60 years completed a survey on COVID-19 impacts in April-May 2020. Compared to before COVID‐19, over 40% of caregivers reported doing worse financially, 16% were experiencing new financial hardship, 19.4% were a lot more worried about their financial situation, 15% reported drinking more alcohol, and 64% had somewhat or greatly increased feelings of social isolation and loneliness. CGs reported that COVID‐19 had made caregiving more physically (18.7%), emotionally (48.5%) and financially (14.5%) difficult and interfered with their own healthcare (19%). Differences found between younger and older caregivers will be presented and implications of these increased CG-related EM risk factors for healthcare and social service providers discussed.

